# Assessment of risk from food allergens cross-contamination

**DOI:** 10.1186/2045-7022-1-S1-S40

**Published:** 2011-08-12

**Authors:** Anton Alldrick

**Affiliations:** 1 Campden BRI, Science Division, Chipping Campden, UK

## 

Successful food-safety management relies on a clear identification of the hazards to be addressed. In the case of issues relating to food allergens the hazard may be defined as: The inadvertant consumption of a food allergen by a sensitive individual. Ensuring that the risk (probability) of this hazard occuring is maintained at an appropriately low level within the context of modern food-manufacturing often presents issues. These reflect the facts that modern food-processing businesses rely on the efficient use of both equipment and the associated manufacturing environment. Such reliance often predicates against the dedicated use of a particular manufacturing line, still less a manufacturing facility, for a single product. This multiplicity of products is often the source of many of the issues relating to food allergen cross-contaminantion. At a philosophical level, the issue of cross contamination presents a unique hazard in terms of food safety management since the contaminant is an integral part of a food and considered nutritious to most consumers. Furthermore levels of cross contamination capable of eliciting an adverse reaction are often lower than those associated with loss of consumer acceptability. Risk assessment cannot seen be seen in isolation and has to be considered within the additional context of risk management and risk communication. Risk management therefore requires an understanding but of both events taking place within the manufacturing facility but also within the suppliers of the raw materials used to make the finished product. As such successful management of food allergen issues places heavy reliance on pre-requisite programmes operating within the food business (e.g. sanitation, training and supplier quality assurance). Finally it is necessary to ensure that relevant information concerning any remaining hazards presented to the food allergic consumer are clearly communicated on the wrapper.

